# Metformin Restores Parkin-Mediated Mitophagy, Suppressed by Cytosolic p53

**DOI:** 10.3390/ijms17010122

**Published:** 2016-01-16

**Authors:** Young Mi Song, Woo Kyung Lee, Yong-ho Lee, Eun Seok Kang, Bong-Soo Cha, Byung-Wan Lee

**Affiliations:** 1 Brain Korea 21 PLUS Project for Medical Science, Yonsei University College of Medicine, 03722 Seoul, Korea; ymsong1225@gmail.com; 2 Division of Endocrinology and Metabolism, Department of Internal Medicine, Yonsei University College of Medicine, 03722 Seoul, Korea; lewoky7@yuhs.ac (W.K.L.); yholee@yuhs.ac (Y.L.); edgo@yuhs.ac (E.S.K.); bscha@yuhs.ac (B.S.C.)

**Keywords:** mitophagy, p53, Parkin, mitochondrial spheroid, metformin

## Abstract

Metformin is known to alleviate hepatosteatosis by inducing 5’ adenosine monophosphate (AMP)-kinase-independent, sirtuin 1 (SIRT1)-mediated autophagy. Dysfunctional mitophagy in response to glucolipotoxicities might play an important role in hepatosteatosis. Here, we investigated the mechanism by which metformin induces mitophagy through restoration of the suppressed Parkin-mediated mitophagy. To this end, our *ob*/*ob* mice were divided into three groups: (1) *ad libitum* feeding of a standard chow diet; (2) intraperitoneal injections of metformin 300 mg/kg; and (3) 3 g/day caloric restriction (CR). HepG2 cells were treated with palmitate (PA) plus high glucose in the absence or presence of metformin. We detected enhanced mitophagy in *ob*/*ob* mice treated with metformin or CR, whereas mitochondrial spheroids were observed in mice fed *ad libitum*. Metabolically stressed *ob*/*ob* mice and PA-treated HepG2 cells showed an increase in expression of endoplasmic reticulum (ER) stress markers and cytosolic p53. Cytosolic p53 inhibited mitophagy by disturbing the mitochondrial translocation of Parkin, as demonstrated by immunoprecipitation. However, metformin decreased ER stress and p53 expression, resulting in induction of Parkin-mediated mitophagy. Furthermore, pifithrin-α, a specific inhibitor of p53, increased mitochondrial incorporation into autophagosomes. Taken together, these results indicate that metformin treatment facilitates Parkin-mediated mitophagy rather than mitochondrial spheroid formation by decreasing the inhibitory interaction with cytosolic p53 and increasing degradation of mitofusins.

## 1. Introduction

A thorough understanding of the molecular machineries involved in the underlying pathophysiology and pharmacodynamics are crucial to the treatment of metabolic disorders including fatty liver disease. We recently elucidated a novel lipophagic mechanism by which metformin, a kind of anti-diabetic drug that disturbs mitochondrial complex I of the electron transport chain, alleviates non-alcoholic fatty liver disease (NAFLD) by inducing the sirtuin class of histone/protein deacetylases sirtuin 1 (SIRT1)-mediated autophagy independent of 5′ adenosine monophosphate-activated protein kinase (AMP-activated protein kinase) [1]. Of the intracellular organelles involved in this underlying pathophysiologic mechanism, the degradation of dysfunctional mitochondria and the reciprocal biogenesis of mitochondria, *i.e.*, mitophagy, are areas of debate and investigation [2,3]. Although controversies remain as to the distinct machinery utilized for mitophagy in response to cell stressors, dysfunctional mitophagy in response to glucose and lipid toxicities in hepatocytes might play an important role in the accumulation of intracellular or extracellular oxidative stress, which causes NAFLD, ultimately leading to hepatocellular carcinoma [3,4,5]. In addition, recent studies have reported that cytosolic p53 interferes with mitochondrial integrity and mitophagy [6,7] by inhibiting the removal of damaged mitochondria through an inhibitory interaction with Parkin, thereby inducing mitochondrial dysfunction [8,9]. Based on these previous reports, we hypothesized and aimed to elucidate the mechanism by which metformin induces mitophagy through restoration of the Parkin-mediated mitophagy suppressed by increased cytosolic ER stress and p53.

## 2. Results

### 2.1. Metformin Treatment Upregulates Mitophagy

**Figure 1:**
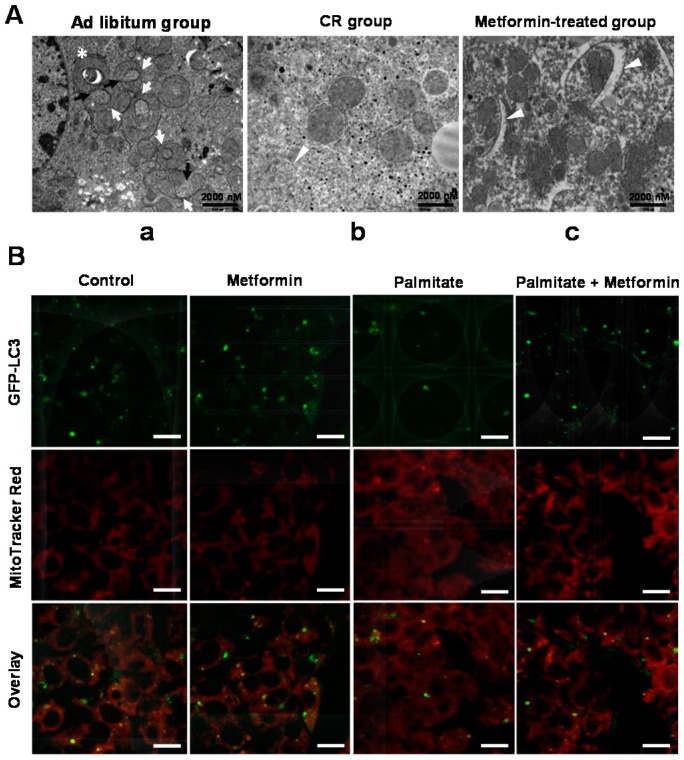
Metformin induced mitophagy. Electron microscopy was performed on hepatocytes from *ob*/*ob* mice under *ad libitum* feeding of chow diet (**A**-**a**), caloric restriction (**A**-**b**), and *ad libitum* feeding of chow diet with 300 mg/kg metformin treatment (**A**-**c**). In the *ad libitum* group, spheroid mitochondria were readily detected. However, in the metformin-treated group, autophagic double membranes were detected, indicating mitophagy; (**B**) co-localization between a marker of autophagosomes (LC3) and a mitochondrial marker (MitoTracker Red) was assessed in HepG2 cells stably transfected with green fluorescent protein (GFP)-LC3. Although there was little co-localization between mitochondria and autophagosomes in the 0.25 mM palmitate-treated HepG2 cells, LC3-labeled structures were seen surrounding the fragmented mitochondria in cells treated with 0.5 mM metformin and 0.25 mM palmitate. Scale bar = 20 μm.

The electron microscopic findings resulting from *ad libitum* feeding of chow diets (Figure 1A-a), caloric restriction (CR) (Figure 1A-b), and *ad libitum* feeding of chow diets and 300 mg/kg metformin treatment (Figure 1A-c) in mice are shown. Electron microscopy readily detected spheroid mitochondria forming a clearly defined internal space surrounded by mitochondrial membranes in mice fed a standard chow diet (Figure 1A-a). The compressed mitochondria (white arrow), the lost cristae, and some portion of the matrix might connect the extra-mitochondrial space through a small orifice (black arrow) or form a lumen containing subcellular contents (asterisk). The white arrowheads indicate either autophagic double membranes surrounding abnormal mitochondria or an autophagic vacuole containing mitochondria (Figure 1A-c).

We assessed co-localization between a marker of autophagosomes, LC3, and a mitochondrial marker (MitoTracker Red) in HepG2 cells stably transfected with GFP-LC3. HepG2 cells ectopically expressing GFP-LC3 exhibited an increase in the number of punctate GFP-LC3 structures upon exposure to 0.5 mM metformin. Furthermore, LC3-labeled structures were found surrounding the fragmented mitochondria in cells treated with metformin and palmitate, although there was little co-localization between mitochondria and autophagosomes in cells treated with 0.25 mM palmitate alone (Figure 1B).

### 2.2. Palmitate Increases but Metformin Decreases the Expression of p53 Protein

**Figure 2:**
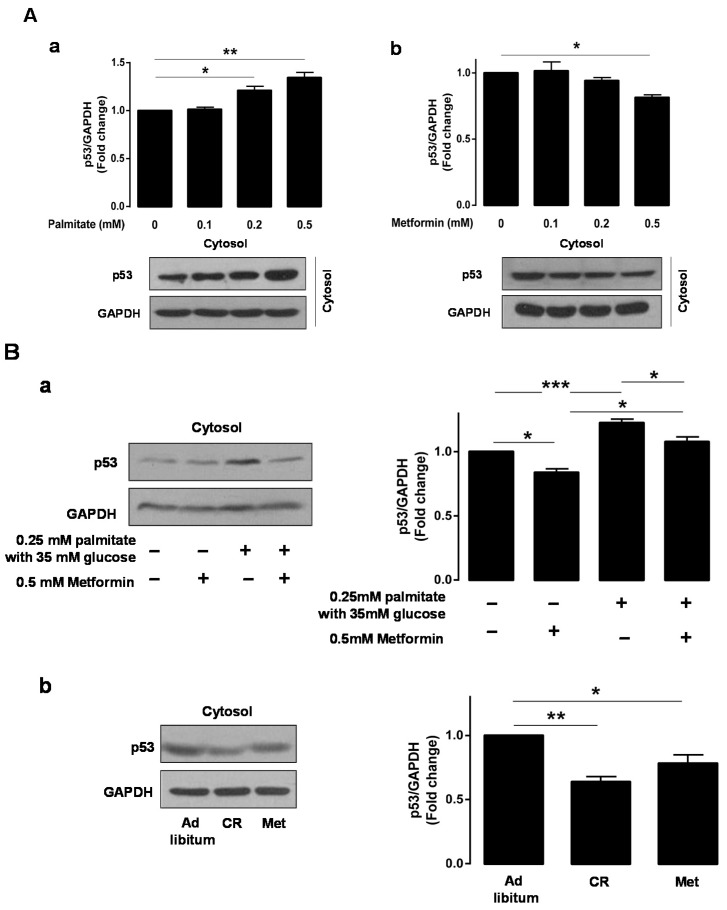

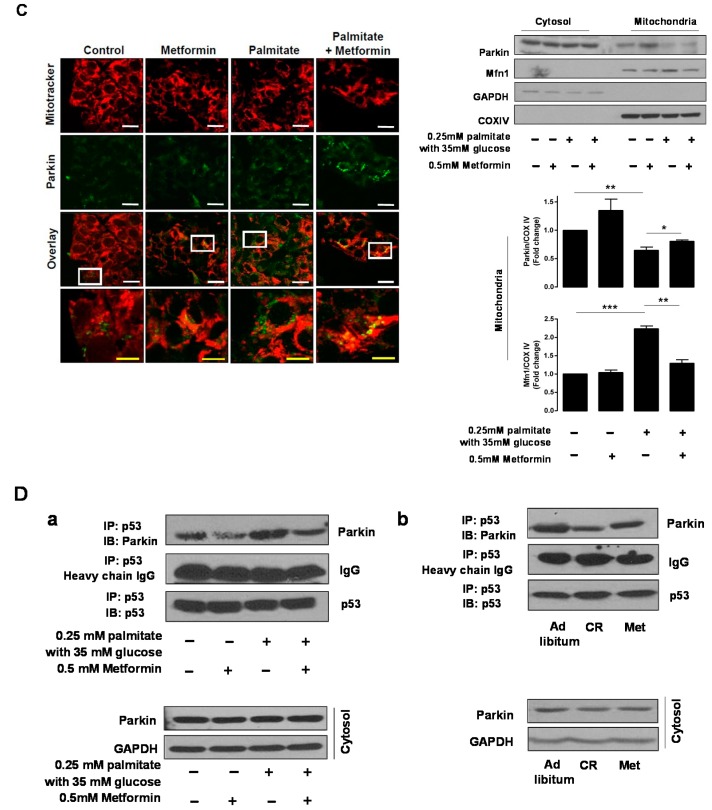
Metformin restored the Parkin-mediated mitophagy inhibited by glucolipotoxicity-induced cytosolic p53. Western blotting analysis was performed to assess cytosolic p53 levels with the cytosolic fractions isolated from palmitate- (**A**-**a**) or metformin- (**A**-**b**) and 35 mM glucose-treated HepG2 cells; the expression of cytosolic p53 protein was assessed following 0.5 mM metformin and/or 0.25 mM palmitate with 35 mM glucose treatment of HepG2 cells (**B**-**a**) or in hepatocytes from *ob*/*ob* mice with *ad libitum* feeding of chow diet, caloric restriction, and *ad libitum* feeding of chow diet with 300 mg/kg metformin treatment (**B**-**b**); (**C**) Expression of Parkin (green) on mitochondria (MitoTracker Red) was observed with a confocal microscopy. The bottom panels show enlarged views of the boxed areas. The yellow dots indicate the mitochondria that co-localize with Parkin. The cytosolic and mitochondrial fractionation experiments demonstrated Parkin translocation from the cytosol to mitochondria depending on the conditions used in the HepG2 cells; a co-immunoprecipitation assay was conducted to investigate the interaction between p53 and Parkin, depending on the conditions used in the HepG2 cells (**D**-**a**) or in hepatocytes from mice (**D**-**b**). GAPDH or COX IV was used for normalization. Values displayed are mean ± SEM (*n* = 3 independent experiments, respectively). Scale bar: white = 20 μm, yellow = 10 μm. Asterisks (* *p* < 0.05, ** *p* < 0.01, *** *p* < 0.001) indicate significant differences. Abbreviation: Mfn1, mitofusin1.

To determine whether metformin down-regulated p53 expression, Western blotting was performed using the cytosolic fraction. Western blot analysis showed a dose-dependent increase in cytosolic p53 protein in response to palmitate in the presence of 35 mM glucose in HepG2 cells (Figure 2A-a). In contrast, when metformin was added to the same conditions, p53 expression decreased in a dose-dependent manner (Figure 2A-b). Treatment of HepG2 cells with both 0.25 mM palmitate and 35 mM glucose significantly activated cytosolic p53 (1.0 ± 0 *vs.* 1.226 ± 0.028, *p* < 0.0001). However, pretreatment with 0.5 mM metformin significantly attenuated this gluco-lipid induced expression of p53 in HepG2 cells (1.226 ± 0.028 *vs.* 1.078 ± 0.038, *p* < 0.05, Figure 2B-a). To confirm the effects of metformin in an animal model, we performed Western blotting using hepatocytes from *ob*/*ob* mice. As shown in Figure 2B-b, expression of p53 was significantly attenuated in both the CR- (0.639 ± 0.072, *p* < 0.001) and metformin-treated groups (0.783 ± 0.114, *p* < 0.05) compared to the *ad libitum*-fed group.

### 2.3. Metformin Induces Parkin-Mediated Mitophagy through Inhibition of Glucolipotoxicity-Induced Cytosolic p53

To highlight the impact of metformin on mitophagy, we assessed co-localization between Parkin and a mitochondrial marker (MitoTracker Red) using immunofluorescence staining, and performed cytosolic and mitochondrial fractionation experiments in HepG2 cells. In the immunofluorescence study, there was little co-localization between Parkin and mitochondria in cells treated with 0.25 mM palmitate and 35 mM glucose, whereas metformin restored the co-localization (yellow dots) between Parkin and mitochondria. In the fractionation experiments, Parkin translocation from the cytosol to mitochondria was attenuated in cells treated with 0.25 mM palmitate and 35 mM glucose, whereas metformin restored the Parkin translocation suppressed by glucolipotoxicity (Figure 2C). We also found a negative relationship between Parkin and mitofusin translocation in response to metformin treatment. To examine p53 and Parkin protein–protein interactions, the endogenous Parkin–p53 complex was observed in immunoprecipitates (IP) of Parkin and p53 (Figure 2D). Treatment of HepG2 cells with both 0.25 mM palmitate and 35 mM glucose significantly upregulated the expression of Parkin. However, pretreatment of HepG2 cells with 0.5 mM metformin significantly attenuated the gluco-lipid induced expression of Parkin (Figure 2D-a); similar findings were also found using *ad libitum*-fed *ob*/*ob* mice and *ob*/*ob* mice treated with CR or metformin (Figure 2D-b). These results indicate the involvement of p53 in the significant decrease in Parkin-dependent mitophagy observed under diabetic conditions.

### 2.4. Metformin Induces Parkin-Mediated Mitophagy through Inhibition of Glucolipotoxicity-Induced Endoplasmic Reticulum (ER) Stress

**Figure 3:**
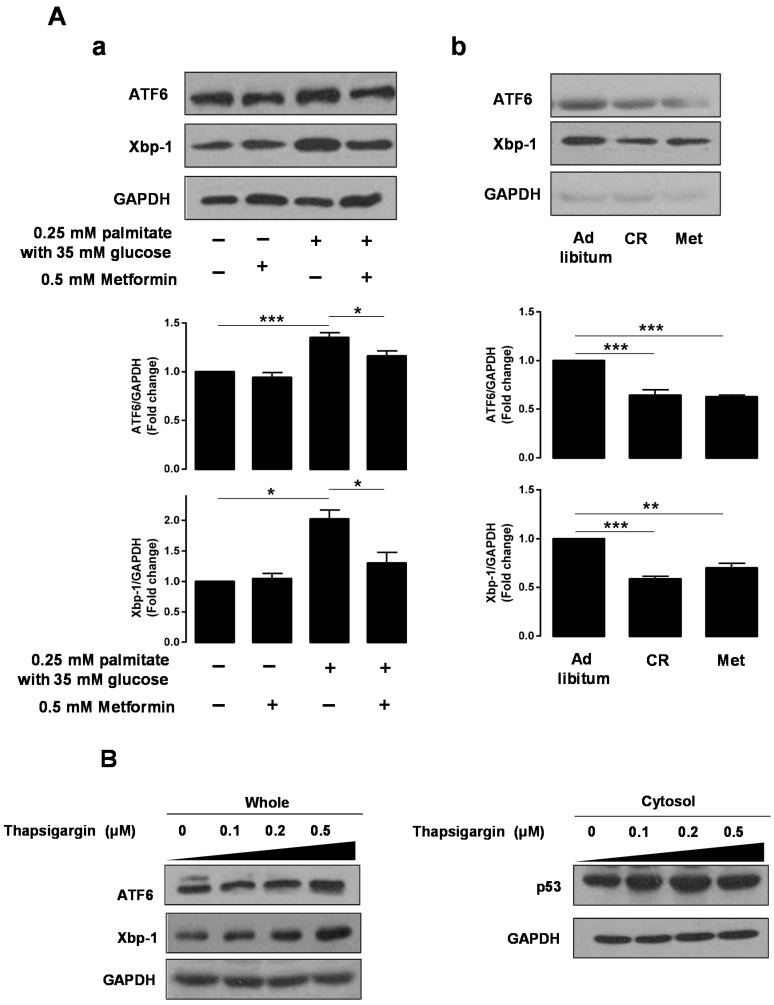

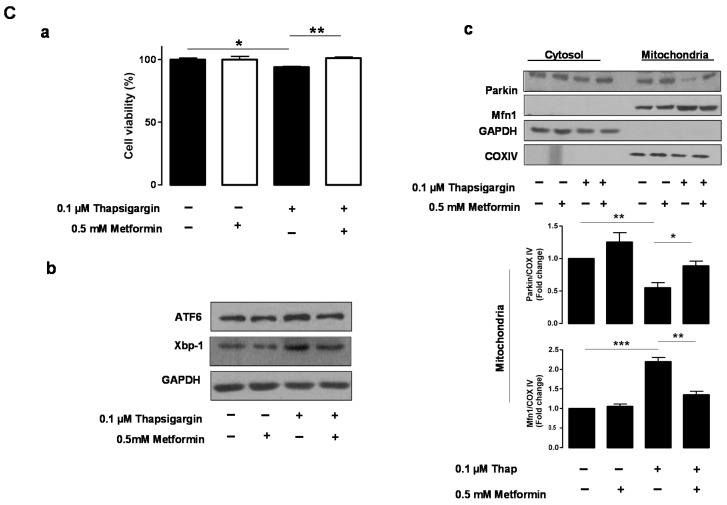
Metformin restored Parkin-mediated mitophagy inhibited by glucolipotoxicity-induced ER stress. The expression of ER stress markers was assessed following 0.5 mM metformin and/or 0.25 mM palmitate with 35 mM glucose treatment of HepG2 cells (**A**-**a**) or in hepatocytes from *ob*/*ob* mice with *ad libitum* feeding of chow diet, caloric restriction, or *ad libitum* feeding of chow diet with 300 mg/kg metformin treatment (**A**-**b**) using Western blotting analysis. Values displayed are mean ± SEM (*n* = 3 independent experiments, respectively); (**B**) the expression of ER stress markers and p53 protein was assessed in response to thapsigargin in HepG2 cells; (**C**-**a**) cell viability was assessed in HepG2 cells exposed to 0.1 μM thapsigargin and/or 0.5 mM metformin using CCK-8. Values displayed are mean ± SEM (*n* = 5 independent experiments); (**C**-**b**) the expression of an ER stress marker was also examined under these conditions; (**C**-**c**) the cytosolic and mitochondrial fractionation experiments demonstrated Parkin translocation from the cytosol to mitochondria, depending on the conditions used. Values displayed are mean ± SEM (*n* = 3 independent experiments, respectively). GAPDH or COX IV was used for normalization. Asterisks (* *p* < 0.05, ** *p* < 0.01, *** *p* < 0.001) indicate significant differences. Abbreviation: Mfn1, mitofusin1; CCK-8, cell counting kit-8.

To elucidate possible ER stress-mediated glucolipotoxicity in a hepatocyte cell line, we investigated the effects of palmitate with glucose and metformin on ER stress markers using Western blotting. We demonstrated that HepG2 cells treated with both 0.25 mM palmitate and 35 mM glucose for 24 h showed significant activation of the expression of ATF6 and Xbp-1. However, pretreatment with 0.5 mM metformin attenuated the expression of ER stress markers in HepG2 cells (Figure 3A-a). Similar findings were noted in both the CR- and metformin-treated groups compared to the *ad libitum*-fed group (Figure 3A-b). In addition, we investigated this pathway using the traditional ER stressor thapsigargin. As shown in Figure 3B, HepG2 cells exposed to different concentrations (0, 0.1, 0.2, and 0.5 μM) of thapsigargin showed upregulated expression of ER stress markers and of p53 protein. Based on this finding, we utilized 0.1 μM thapsigargin in subsequent experiments. Compared to the untreated controls, HepG2 cells exposed to 0.1 μM thapsigargin for 24 h showed significantly decreased levels of viability (100.0 ± 2.95 *vs.* 94.0 ± 1.24, *p* < 0.05) although there is no significant difference in viability in HepG2 cells exposed to 0.5 mM metformin for 24 h, as assessed by CCK-8. Pretreatment with 0.5 mM metformin for 3 h significantly restored the HepG2 cell viability reduced by 0.1 μM thapsigargin (94.0 ± 1.24 *vs.* 101.1 ± 2.18, *p* < 0.001, Figure 3C-a). To elucidate possible ER stress-mediated mitophagic dysfunction, we performed cytosolic and mitochondrial fractionation experiments in HepG2 cells. Parkin translocation from the cytosol to mitochondria was attenuated in cells treated with 0.1 μM thapsigargin, whereas metformin restored the Parkin translocation suppressed by thapsigargin (Figure 3C-b,c).

### 2.5. A p53 Inhibitor Restores Parkin-Mediated Mitophagy Suppressed by Glucolipotoxicity

**Figure 4:**
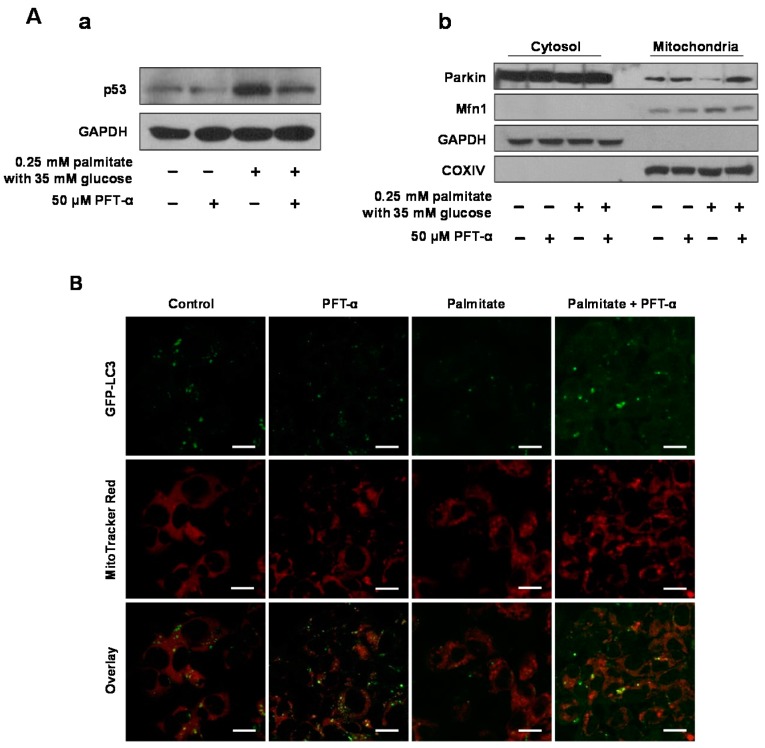
A p53 inhibitor restored the Parkin-mediated mitophagy inhibited by glucolipotoxicity. (**A**-**a**) the expression of cytosolic p53 was assessed in HepG2 cells treated with 50 μM PFT-α, a p53 inhibitor, and/or 0.25 mM palmitate with 35 mM glucose using Western blotting analysis; (**A**-**b**) the cytosolic and mitochondrial fractionation experiments demonstrated Parkin translocation from the cytosol to mitochondria, depending on the conditions used. Exposure time of Western blotting was long, compared to Figure 2C; (**B**) co-localization was assessed between a marker of autophagosomes (LC3) and a mitochondrial marker (MitoTracker Red) in HepG2 cells stably transfected with GFP-LC3, depending on the conditions used. LC3-labeled structures were observed surrounding the fragmented mitochondria in cells treated with PFT-α and palmitate, although there was little co-localization between mitochondria and autophagosomes in cells treated with 0.25 mM palmitate alone. Scale bar = 20 μm. *Abbreviation*: Mfn1, mitofusin1; PFT-α, pifithrin-α.

To determine whether p53 actively mediates mitophagy dysfunction, we treated cells with pifithrin-α (PFT-α), a p53 inhibitor, to act as an ER stress reliever. Parkin translocation from the cytosol to mitochondria was attenuated in cells treated with 0.25 mM palmitate and 35 mM glucose, whereas PFT-α restored the Parkin translocation suppressed by glucolipotoxicity (Figure 4A). We additionally assessed co-localization between a marker of autophagosomes, LC3, and mitochondria in HepG2 cells stably transfected with GFP-LC3. LC3-labeled structures were observed surrounding the fragmented mitochondria in cells treated with PFT-α and palmitate, although there was little co-localization between mitochondria and autophagosomes in cells treated with 0.25 mM palmitate alone. (Figure 4B).

## 3. Discussion

Mitochondria not only produce cellular energy through the oxidation of nutrients, but also generate reactive oxygen species (ROS) under pathologic conditions that induce mitochondrial dysfunction [10]. The generation of ROS in conjunction with endoplasmic reticulum (ER) stress induces cellular stress and, consequently, cell death [11,12]. Because the existence of damaged and aged mitochondria threatens cells, the turnover processes of mitochondrial degradation and biogenesis are essential for maintenance of cellular integrity [2,3,4,13]. With advances in the understanding of autophagy, mitophagy, the selective degradation of mitochondria by autophagy, has garnered attention in mitochondrial metabolism. Conventionally, metformin has been viewed as a type of anti-diabetic drug belonging to the biguanide class that functions by disturbing complex I of the electron transport chain, thereby resulting in activation of 5′ adenosine monophosphate-activated protein kinase (AMP-activated protein kinase) and induction of the activation of NAD+-dependent deacetylase SIRT1 (sirtuin 1) [14,15]. Recently, we demonstrated that metformin activates lipophagy, resulting in relief of hepatosteatosis due to induction of SIRT1-mediated autophagy independent of AMP-activated protein kinase [1]. Based on these reports, we hypothesized that metformin plays additional roles in mitochondrial homeostasis through the activation of mitophagy, resulting in an improvement in hepatosteatosis.

Among the cell stress or apoptotic pathways, p53 is known to utilize many mechanisms in its anticancer function and to play roles in apoptosis and genomic stability. Recent studies have reported that cytosolic p53 contributes to mitochondrial integrity and mitophagy [6,7]. Cytosolic p53 has been shown to inhibit the removal of damaged mitochondria through an inhibitory interaction with Parkin, thereby inducing mitochondrial dysfunction [8,9]. Based on these reports, we studied the interplay of metformin in mitophagy and p53 abundance. We therefore investigated (1) whether metformin alleviates the glucolipotoxicity induced cell stress by assessing the expression of ER stress markers and p53 protein; (2) whether Parkin-mediated mitophagy is inhibited by glucolipotoxicity-induced cytosolic p53; and (3) whether metformin induces mitophagy through restoration of the suppressed Parkin-mediated mitophagy induced by increased cytosolic ER stress and p53.

With respect to glucolipotoxicity-induced hepatocellular toxicity and the role of metformin in alleviating glucolipotoxicity, we observed a dose-dependent increase in p53 protein expression in HepG2 cells treated with palmitate in the presence of 35 mM glucose, as well as a dose-dependent decrease in p53 expression in HepG2 cells treated with metformin. Additionally, we found that metformin alleviated glucolipotoxicity-induced p53 overexpression. Similar to these findings, metformin alleviated glucolipotoxicity-induced ER stress, as assessed by Western blotting using ATF6 and Xbp-1 antibodies. To further demonstrate ER stress pathway-mediated glucolipotoxicity, we treated HepG2 cells with the well-established ER stressor thapsigargin (TG). In these experiments, we observed a dose-dependent upregulation of cytosolic p53 and the ER stress markers ATF6 and Xbp-1 in TG-treated HepG2 cells. From a translational point of view [16], nutritional deprivation through caloric restriction (CR) and metformin administration in *ob*/*ob* mice also showed significantly decreased levels of cytosolic p53 and the ER stress markers ATF6 and Xbp-1 compared to *ad libitum* fed *ob*/*ob* mice. Endoplasmic reticulum (ER) plays a role as a nutrient sensor in cells, and excess fuel can induce ER stress, which triggers the unfolded protein response (UPR). The UPR leads to both translational attenuation of new protein synthesis and transcriptional activation of stress-response genes to relieve ER stress. However, the UPR also initiates death signals, which function when the stress is pathologically prolonged [17]. ER stress has been generally shown to be inhibited by metformin [18,19], thereby preventing cell injury and apoptosis. Based on results in this study, we suggest that increased expression of p53 accompanies the ER stress pathway.

With respect to mitophagy in hepatic cells, *ob*/*ob* mice treated with metformin or CR showed increased mitophagy, as demonstrated by autophagic double membranes surrounding abnormal mitochondria or by the presence of an autophagic vacuole containing mitochondria. In contrast, *ob*/*ob* mice under metabolic stress caused by *ad libitum* feeding of chow diets showed numerous mitochondrial spheroids, as demonstrated by the compressed mitochondrion and lost cristae and matrix. To further validate mitophagy induction by metformin treatment, we additionally demonstrated increased co-localization of the autophagy marker GFP-LC3 and the selective mitochondrial probe MitoTracker Red in metformin-treated HepG2 cells. Mitochondrial spheroids are described as having a unique ring-like morphology with a squeezed mitochondrial matrix. Under various pathophysiologic stresses, mitochondria can undergo direct remodeling to form mitochondrial spheroids, which requires the presence of ROS and either mitofusin1 (Mfn1) or mitofusin2 (Mfn2). In this process, Parkin prevents mitochondrial spheroid formation by causing proteasomal degradation of Mfn1 and Mfn2, which are required for mitophagy [20]. Mitochondrial spheroids can envelop the contents of the cytosol, including damaged mitochondria. Regarding fat accumulation in the liver, each hepatocyte contains about 800 mitochondria, and mitochondrial dysfunction can contribute to the development of NAFLD [21,22,23]. In a recent study, Ibdah *et al.*, using heterozygous mice deficient in mitochondrial trifunctional protein, found that impairment in β-oxidation resulted in hepatic steatosis [24]. These alterations were also demonstrated in Otsuka Long-Evans Tokushima Fatty (OLETF) rats, which is a model of type 2 diabetes and obesity [25]. Based on the results of our study, we suggest that the metformin-induced autophagic machinery might play important roles in alleviating hepatosteatosis through the induction of both lipophagy and mitophagy.

With respect to the inverse relationship between metformin-induced mitophagy induction and p53 protein abundance [26], the present study showed that glucolipotoxicity inhibited the translocation of Parkin from the cytosol to mitochondria. In contrast, we also showed that metformin or PFT-α, which is the p53 inhibitor, induced translocation of Parkin from the cytosol to mitochondria by decreasing the inhibitory interaction with cytosolic p53. These results were additionally validated by examining protein–protein interactions between p53 and Parkin using immunoprecipitates (IP) of endogenous Parkin and p53, as well as the co-localization of autophagy marker GFP-LC3 and the selective mitochondrial probe Mitotracker Red in PFT-α-treated HepG2 cells. Regarding the role of mitofusin in mitochondrial spheroid formation, ROS and either Mfn1 or Mfn2 are required. The inner membrane protein optic atrophy 1 (OPA1) might not be important for this process because it is rapidly degraded upon mitochondrial depolarization. Ding *et al.* reported that Parkin promotes mitophagy through the proteasomal degradation of mitofusins and, consequently, the inhibition of mitochondrial spheroid formation [20]. We confirmed the negative relationship between Parkin and mitofusins in mitochondrial quality control in response to metformin treatment in this study. Based on these results, we suggest that metformin treatment facilitates Parkin-mediated mitophagy instead of mitochondrial spheroid formation by decreasing the inhibitory interaction with cytosolic p53 and increasing the degradation of mitofusins.

**Figure 5:**
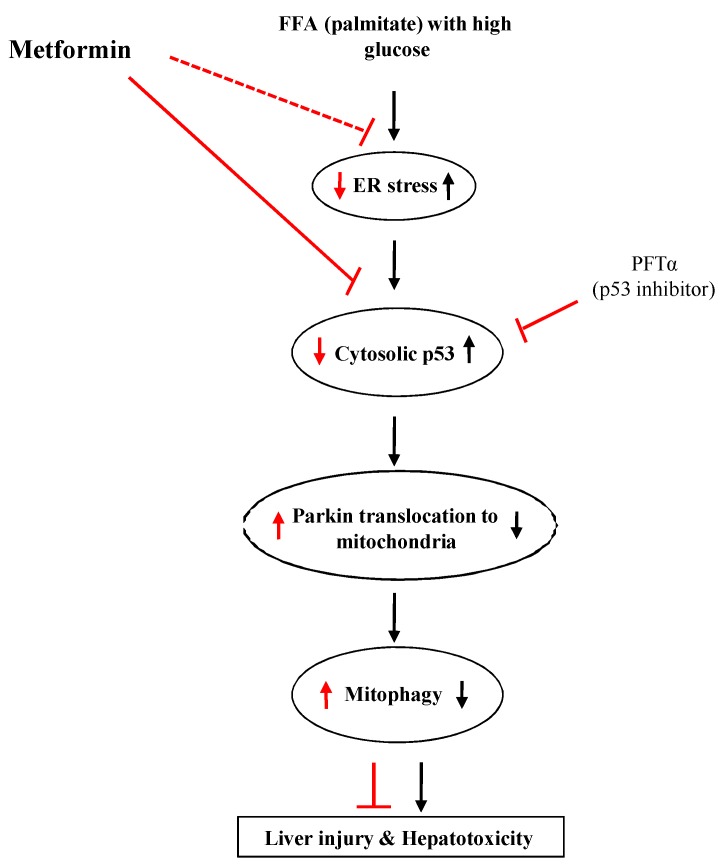
Summary of the working thesis of this study. See the text for details. Black and red arrows indicate the effects of glucolipotoxicity and the drugs (metformin or PFTα), respectively.

The limitation of this study is that we did not conduct *in vivo* experiments with genetically engineered animal models, such as Parkin knockout mice, to provide a more convincing mechanistic role of metformin in mitophagy. Consequently, these studies should be performed in the future. However, to the best of our knowledge, our study is the first to investigate the molecular interplay among glucolipotoxicity-induced ER stress, cytosolic p53, and Parkin-mediated mitophagy in response to metformin treatment. To summarize our findings, metformin treatment might facilitate Parkin-mediated mitophagy instead of mitochondrial spheroid formation by decreasing the inhibitory interaction with cytosolic p53 and increasing the degradation of mitofusins. This working thesis is summarized in Figure 5.

## 4. Experimental Section

### 4.1. Animal and Experimental Procedures

Eight-week-old *ob*/*ob* mice (C57bl/6j background, Jackson Laboratory, Bar Harbor, ME, USA) were maintained at 60% ± 5% relative humidity and 22 ± 2 °C with a 12-h light/dark cycle over a four-week course of the following treatment regimens: (1) *ad libitum* feeding of chow diet (Dyets Inc., Bethlehem, PA, USA) and vehicle injection of PBS (pH 7.8, *n* = 8); (2) *ad libitum* feeding of chow diet and intraperitoneal injections of 300 mg/kg metformin (Merck KGaA, Darmstadt, Germany, *n* = 8); and (3) caloric restriction (CR) group (3 g/day, *n* = 8). After each animal was sacrificed, fresh liver tissues were fixed with 2% glutaraldehyde-2% paraformaldehyde buffered with 0.1 M phosphate buffer (pH 7.2) overnight at 4 °C, post-fixed with 1% osmium tetroxide in a 0.1 M sodium cacodylate buffer (pH 7.4) for 1 h at room temperature, and dehydrated with a graded series of ethanol, as previously described [5]. The dehydrated tissues were then embedded in Epon, using poly/Bed812 Embedding kit/ DMP-30 (Polysciences, Warrington, PA, USA). All experimental procedures performed in this study followed the ethical guidelines for animal studies and were approved by the Institutional Animal Care and Use Committee of Yonsei University College of Medicine (IACUC No. 2011-0302-1).

### 4.2. Cell Culture and Preparation of Subcellular Fractions

The human hepatoma cell line HepG2 was cultured in Dulbecco’s modified Eagle’s medium (Welgene, Daegu, Korea) supplemented with 10% FBS and antibiotics. HepG2 cells were washed and incubated for 10 min in ice-cold PBS, scraped into PBS with 1 mM phenylmethylsulfonylfluoride (PMSF), and pelleted by centrifugation (500× *g*) at 4 °C for 10 min. Cell pellets were resuspended in RIPA buffer (Life Science, Berkeley, CA, USA) with protease inhibitors (Thermo Scientific, Waltham, MA, USA), passed 10 times through a 30-gauge syringe to break open the cells, and incubated on ice for 10 min to generate a whole cell lysate. After centrifugation (10,000× *g*, in a microcentrifuge) at 4 °C for 30 min, the supernatants were collected as whole extracts. Fractionation to separate the mitochondrial and cytosolic fractions was carried out as previously described [27]. Briefly, HepG2 cells were harvested, washed in PBS, and resuspended in cytosolic extraction buffer (250 mM sucrose, 70 mM KCl, 137 mM NaCl, 4.3 mM Na_2_HPO_4_, 1.4 mM KH_2_PO_4_, pH 7.2, 200 μg/mL digitonin, 100 μM PMSF, protease inhibitor cocktail) (Sigma, St. Louis, MO, USA) for 5 min on ice. Digitonin-permeabilized cells were confirmed by staining with 0.2% trypan blue solution. Cells were centrifuged at 1000× *g* for 5 min; the supernatant was saved as the cytosolic fraction, and the pellet was solubilized in two volumes of mitochondrial lysis buffer (50 mM Tris-HCl pH 7.4, 150 mM NaCl, 2 mM EDTA, 2 mM EGTA, 0.2% (*v*/*v*) Triton X-100, 0.3% NP-40, PMSF, protease inhibitor cocktail) (Sigma) for 5 min on ice. Cells were then centrifuged at 10,000× *g* for 10 min at 4 °C, and the supernatant was collected as the mitochondrial extract.

### 4.3. Detection of Mitophagy

For the detection of autophagosomes and mitochondrial spheroids using electron microscopy, sectioning for electron microscopic examination was accomplished with an ultramicrotome (Leica, EM UC7, Wetzlar, Germany), and electron microscopy was performed with a JEM-1011 transmission electron microscope (JEOL). Detection of mitophagy were also performed by transfecting HepG2 cells with a plasmid driving the expression of GFP-LC3 (excitation wavelength 488 nm, emission filter 530 nm) (OriGene Technologies, Inc., Rockville, MD, USA) for 16 h, followed by loading of cells with Mitotracker Red (500 nM) (excitation wavelength 488 nm, emission filter 585 nm) (Invitrogen, Carlsbad, CA, USA). Transfected cells were fixed with 4% paraformaldehyde and then observed with a confocal microscope.

### 4.4. Immunofluorescence Staining

To detect Parkin expression on mitochondria, immunofluorescence staining was performed on cells using Parkin antibody and MitoTracker Red. The cells were stained with MitoTracker Red (500 nM) and fixed with 4% paraformaldehyde. The fixed cells were incubated with Parkin antibody for 2 h, followed by incubation with secondary antibody (Alexa 488, excitation wavelength 495 nm, emission filter 519 nm) (Invitrogen) for 2 h. Next, the cells were observed under the confocal microscope.

### 4.5. Free Fatty Acid (FFA) Preparation

FFA solutions were prepared as previously described [15]. Briefly, 100 mM palmitate (PA) (Sigma) stocks were prepared in 0.1 M NaOH at 70 °C and filtered. A one percent (weight/volume) palmitate-free BSA (Sigma) solution was prepared in serum-free DMEM. After the palmitate dissolved, the palmitate solutions were added to serum-free DMEM containing BSA. The 5 mM palmitate/1% BSA solution was prepared by complexing the appropriate amounts of palmitate to 1% BSA in a 55 °C water bath.

### 4.6. Cell Viability Assay

HepG2 cells were dispensed in wells of 24-well plates at a density of 5 × 10^4^ cells/well. HepG2 cells were pretreated with 0.5 mM metformin for 3 h and incubated in the presence or absence of thapsigargin for 24 h. The cells were then treated with 10 μL of cell counting kit-8 (CCK-8) solution (Sigma-Aldrich, St. Louis, MO, USA) at 37 °C for the indicated times, according to the manufacturer’s instructions. Absorbance was measured at 450 nm using a microplate reader (Molecular Devices, Sunnyvale, CA, USA).

### 4.7. Western Blotting and Antibodies

Mouse livers and HepG2 cells were lysed in PRO-PREPTM protein extraction solution (iNtRON Biotechnology, Kyungki-Do, Korea), and the protein contents of the resulting lysates were measured using the Bradford assay (Bio-Rad, Berkeley, CA, USA). Equal amounts of protein were resolved using SDS-PAGE and were electroblotted onto a nitrocellulose membrane (Bio-Rad). Membranes were subsequently probed as indicated with the following primary antibodies: p53 (Cell Signaling, Danvers, MA, USA), ATF6, sXBP1, GAPDH, Parkin, mitofusin (Mfn1) (Santa Cruz Biotechnology, Santa Cruz, CA, USA), and COXVI (Life Science). Secondary antibodies (anti-rabbit and anti-mouse) were from Santa Cruz Biotechnology.

### 4.8. Co-Immunoprecipitation Assay

For co-immunoprecipitation (IP) assays, HepG2 cells and liver tissue were harvested in lysis buffer (50 mM Tris (pH 7.4), 140 mM NaCl, 1% Triton X-100, 30 M MG132, and protease inhibitors). Cell extracts were centrifuged, and the supernatants were incubated with anti-p53 or anti-Parkin antibodies for 12 h at 4 °C. ProteinA/G PLUS-Agarose (Santa Cruz Biotechnology) was then added to each sample, and incubation was carried out overnight at 4 °C on a rotating device. Immunoprecipitates were collected by centrifugation at 1000× *g* for 5 min at 4 °C and washed with lysis buffer containing 500 mM NaCl or PBS. The pellets were eluted by heating at 95 °C for 5 min in 1× electrophoresis sample buffer.

### 4.9. Statistical Analysis

Statistical analysis was performed using PRISM (GraphPad Software Inc., San Diego, CA, USA). Results are expressed as mean ± SE, and statistical significance was calculated using Student’s *t*-test. Alternatively, for comparisons involving more than two groups, one-way analysis of variance (ANOVA) with a post hoc Bonferroni multiple comparison test was used to assess the differences. Statistical significance was defined as the conventional *p*-value of <0.05.

## 5. Conclusions

In conclusion, the present study suggests that metabolic stresses in *ob*/*ob* mice induce mitochondrial spheroid formation and ultimately result in fat droplet accumulation in hepatocytes. In contrast, metformin treatment of *ob*/*ob* mice induces Parkin-mediated mitophagy upregulation by inhibiting glucolipotoxicity-induced cytosolic p53.
